# Diploids in the *Cryptococcus neoformans* Serotype A Population Homozygous for the α Mating Type Originate via Unisexual Mating

**DOI:** 10.1371/journal.ppat.1000283

**Published:** 2009-01-30

**Authors:** Xiaorong Lin, Sweta Patel, Anastasia P. Litvintseva, Anna Floyd, Thomas G. Mitchell, Joseph Heitman

**Affiliations:** Department of Molecular Genetics and Microbiology, Duke University Medical Center, Durham, North Carolina, United States of America; University of Melbourne, Australia

## Abstract

The ubiquitous environmental human pathogen *Cryptococcus neoformans* is traditionally considered a haploid fungus with a bipolar mating system. In nature, the α mating type is overwhelmingly predominant over **a**. How genetic diversity is generated and maintained by this heterothallic fungus in a largely unisexual α population is unclear. Recently it was discovered that *C. neoformans* can undergo same-sex mating under laboratory conditions generating both diploid intermediates and haploid recombinant progeny. Same-sex mating (α-α) also occurs in nature as evidenced by the existence of natural diploid αADα hybrids that arose by fusion between two α cells of different serotypes (A and D). How significantly this novel sexual style contributes to genetic diversity of the *Cryptococcus* population was unknown. In this study, ∼500 natural *C. neoformans* isolates were tested for ploidy and close to 8% were found to be diploid by fluorescence flow cytometry analysis. The majority of these diploids were serotype A isolates with two copies of the α *MAT* locus allele. Among those, several are intra-varietal allodiploid hybrids produced by fusion of two genetically distinct α cells through same-sex mating. The majority, however, are autodiploids that harbor two seemingly identical copies of the genome and arose via either endoreplication or clonal mating. The diploids identified were isolated from different geographic locations and varied genotypically and phenotypically, indicating independent non-clonal origins. The present study demonstrates that unisexual mating produces diploid isolates of *C. neoformans* in nature, giving rise to populations of hybrids and mixed ploidy. Our findings underscore the importance of same-sex mating in shaping the current population structure of this important human pathogenic fungus, with implications for mechanisms of selfing and inbreeding in other microbial pathogens.

## Author Summary

Although sex typically involves partners of opposite mating type (sexuality), it can also occur with just one mating type and even single individuals (parthenogenesis, homothallism). However, from a population perspective, sexual reproduction occurs by either outcrossing or inbreeding regardless of the partners' sexuality. Here the impact of sex was studied for *Cryptococcus neoformans*, a pathogen that causes fungal meningitis. While sex in the laboratory is known to occur via opposite-sex-mating, the population is largely unisexual (α) in nature. Recently, an unusual α-α unisexual mating process involving only mating type α was discovered in the lab, but the impact of unisexual mating in nature was unknown. The global survey of this typically haploid organism reveals ∼8% diploids in the population produced by unisexual α-α mating. Some diploids result from fusion of two genetically distinct parents while other diploids arise via sibling mating or genome duplication. Although hybrid fitness is well-documented, how sex between identical isolates benefits the population is a conundrum. The diploid state may confer growth advantages or serve as a capacitor for evolution, allowing mutations to arise that would be deleterious on their own in the haploid, and then releasing these in novel combinations by meiosis and sporulation.

## Introduction

Polyploidization and hybridization are driving forces for the generation of genetic diversity in eukaryotic organisms, including pathogens [1]–[4]. These processes are intertwined events as polyploidization often arises through hybridization. Polyploidization is prominent in the plant kingdom, where ploidy is a key determinant of fertility and morphological and quantitative traits. About 10% of plant species harbor individuals of distinct ploidies in addition to the normal diploid [5]. For example, rose species and their hybrids can have a ploidy of 2n, 3n, 4n, 5n, and 6n. Wheat is a polyploid species that originated by hybridization of two or three different diploid species [6]. The evolutionary advantage of polyploidy is evident by the fact that natural polyploid species represent more than 70% of plant species [7].

Many fungal species are predominantly haploid. Although mixed ploidy in a fungal population is considered unusual in the fungal kingdom, limited studies evaluating ploidy variations in natural fungal populations suggest that polyploidy may be more common among fungi. For example, natural populations of the well-studied yeast *S. cerevisiae* revealed considerable variation in ploidy [8]. *Candida albicans*, a human commensal and pathogen, also shows varied ploidy [1],[9],[10]. *Paracoccidioides brasiliensis*, the etiological agent of the most important systemic mycosis in Latin America (paracoccidioidomycosis), exhibits extensive colonial plasticity and phenotypic variation. There is some indication that *P. brasiliensis* may exist both as haploid and diploid [11], and some phenotypic differences are associated with the ploidy level of the strains [12],[13].

Cryptococcal meningitis is the most common fungal infection of the central nervous system and is considered an AIDS defining condition [14]. It is particularly prevalent and devastating in southeast Asia and Africa, where it can cause up to 44% mortality in AIDS patients due to its nearly uniform lethality without timely and proper treatment [15]–[18]. The causative fungus *Cryptococcus neoformans* is ubiquitous in the environment and infects humans and other animals through inhalation. It is an encapsulated yeast with three serotypes based on capsular epitopes: serotype A (*C. neoformans* var. *grubii*), serotype D (*C. neoformans* var. *neoformans*), and serotype AD (hybrid) [19]. Genome sequences of the serotype A and D reference strains H99 and JEC21 reveal that the two serotypes harbor ∼10–15% nucleotide polymorphisms at the genomic level [20],[21]. Despite their divergence at the nucleotide level, isolates of the two serotypes can still mate with each other under laboratory conditions, albeit with reduced ability to produce viable meiotic progeny due to their genetic divergence possibly indicative of cryptic speciation process [22]. In addition, AD hybrids are commonly present in the environment and clinical samples [23], and hybridization events between isolates of serotype A and D are likely ongoing [23]–[26]. Thus, serotype A and D represent two separate varieties in the same species. Serotype A is the most common serotype and is responsible for over 95% of cryptococcosis worldwide [14]. *C. neoformans* can be further classified into at least five distinct molecular types based on DNA sequence polymorphisms detected by PCR fingerprinting, randomly amplified polymorphic DNA (RAPD), amplified fragment length polymorphism (AFLP), restriction fragment length polymorphism (RFLP), and multilocus sequence typing (MLST) analyses [25],[27],[28]. Serotype A isolates include molecular types VNI, VNII, and VNB [25], serotype D isolates belong to molecular type VNIV, and AD hybrids are molecular type VNIII.

*C. neoformans* contains one mating type locus, which is analogous to the S locus in plants and the sex chromosomes of animals. As a bipolar mating system, the mating type locus of *C. neoformans* occurs in either of two alleles, **a** or α [29],[30]. Conventional **a**-α mating leading to the production of meiotic haploid progeny has been observed for isolates of *C. neoformans* serotype A and D, and the sibling species *C. gattii* under laboratory conditions [29]–[32]. *C. neoformans* had been thus considered a fungus with a typical bipolar heterothallic mating system where compatible mating type partners (α and **a** cells) are required for sexual reproduction to occur [33]. Because the overwhelming majority of clinical and environmental isolates of *C. neoformans* have the α mating type (*MAT*α) (>98–99.9%) [14],[34], it is unlikely that conventional **a**-α mating is the only means by which genetic diversity is generated in nature.

The global population structure of *C. neoformans* and *C. gattii* is largely clonal [25], [35]–[37]. However, population genetic studies indicate a low level of recombination in [25], [38]–[44]. Haploid serotype D strains, especially α cells, are able to produce stable diploid cells and haploid meiotic spores through a same-sex mating process under laboratory conditions [45]. Besides indirect evidence from population genetic studies that support the occurrence of same-sex mating in natural populations [38],[41],[43], the discovery of αADα hybrids that arose by fusion between two α cells of serotype A and D indicate that same-sex mating indeed occurs in nature [46]. However, the frequency of the α-α mating and the impact of this mating mode to the population structure of the most virulent serotype A isolates were unknown. Based on our previous observations, we hypothesized that diploid α/α isolates would be present in the largely unisexual *Cryptococcus* serotype A population.

Diploid *C. neoformans* cells of α mating type with an unknown origin have been reported previously [47]–[50]. If diploids are common in the population of *C. neoformans*, ploidy shifts could play an important role in its life cycle in nature. Access to both the haploid and the diploid state may provide a greater range of adaptability than either ploidy alone and may also contribute to the evolution and maintenance of fitness and virulence in this fungus [51]–[54]. However, despite abundant information about the distribution of mating types and serotypes in the global population of *C. neoformans*, there is limited information about ploidy distribution (reviewed by [14],[19]).

In plants, autopolyploids (two identical genomes) arise at a much higher rate than allopolyploids (two different genomes) [55]–[57]. However, allopolyploids are more likely to be recognized compared to autopolyploids because autopolyploids may be phenotypically more similar to their progenitors of lower ploidy [56]. Similarly, cryptic natural diploid αAAα strains of *C. neoformans* may represent a significant proportion of the *Cryptococcus* population. To investigate this issue, we determined the ploidy of a large number of global *Cryptococcus* isolates and found that close to 8% of *Cryptococcus* isolates are diploid. A combination of AFLP, MLST, and comparative genome hybridization (CGH) analyses revealed that a majority of these natural diploids were serotype A isolates with two copies of the α mating type locus allele that arose via same-sex mating (hybridization or endoreplication). These natural diploid isolates display varied morphology and phenotypes *in vitro*, including capsule production, melanization, growth at extreme temperatures, resistance to UV irradiation, and invasive growth.

The study of the ploidy distribution in the *C. neoformans* population, their mechanisms of diploidization, and the contribution of this life strategy to the current population structure of this pathogen provide insight into the plasticity of fungal life styles. It further provides a paradigm to study the potential impact of similar processes on the evolution of unisexual species.

## Results

### *C. neoformans* population is comprised of a mixture of haploids and diploids

*C. neoformans* is considered a haploid fungus in which the α mating type predominates in nature. Natural diploid isolates do occur as serotype AD inter-varietal hybrids that contain two divergent genomes and are impaired in their ability to undergo meiosis [22], [25], [26], [48], [58]–[60]. We hypothesized that AA or DD intra-varietal hybrid diploid α/α isolates would exist in nature based on the facts that (i) same-sex mating can produce stable diploids under laboratory conditions, (ii) α-α mating occurs in nature as evidenced by the existence of αADα hybrids [45],[46], and (iii) autopolyploids usually arise at a much higher rate than allopolyploids [55]–[57]. To test this hypothesis, the *C. neoformans* population (clinical and environmental isolates) was surveyed.

**Figure 1:**
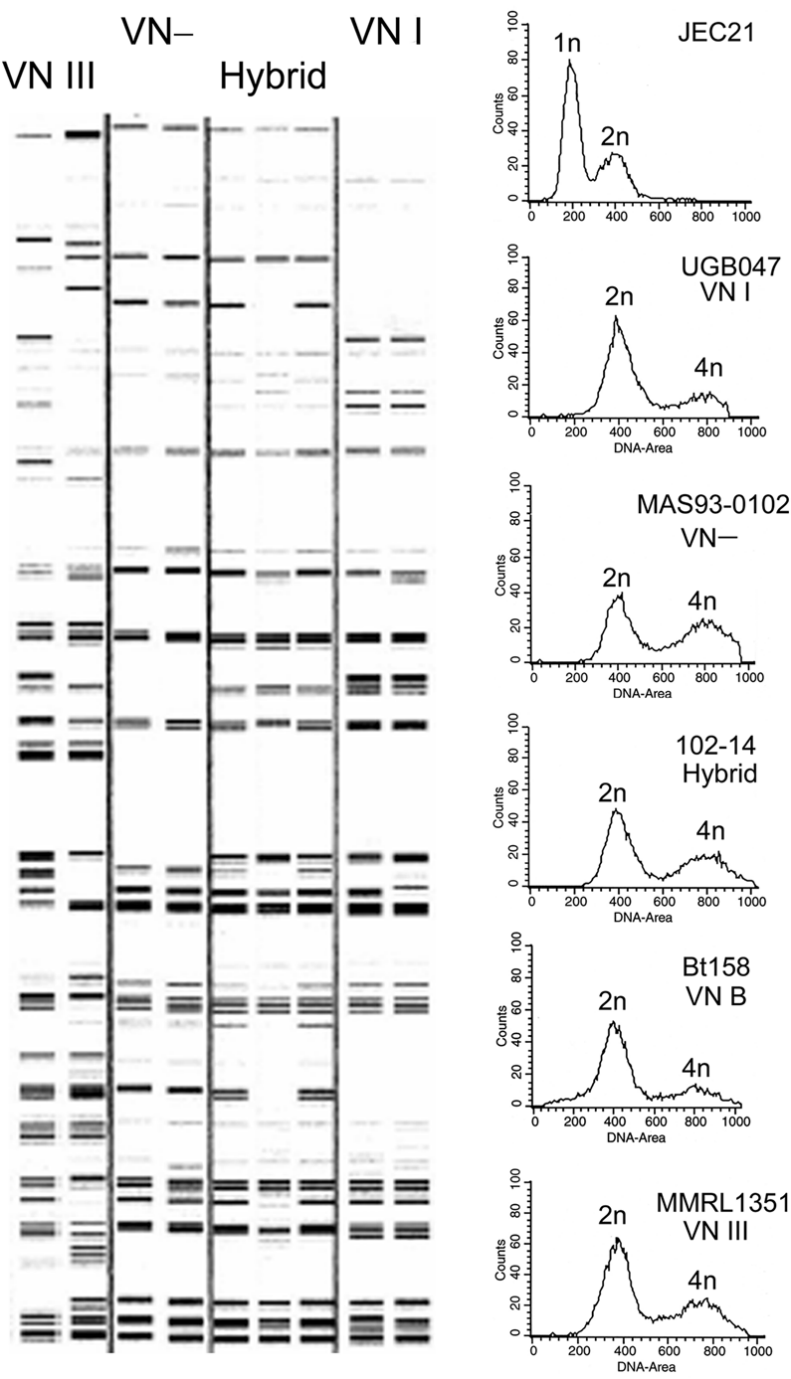
Diploid isolates contain twice the haploid cellular DNA content and belong to different molecular types. Diploid isolates were analyzed by AFLP using primer sets EAC and ETG respectively as previously described [40],[62]. Four molecular types of diploids were identified and the AFLP patterns using the ETG primer set of selected isolates are shown on the left. From left to right are isolates MMRL1351 (VNIII), MMRL752 (VNIII), MAS93-0102 (VNII), MAS92-0333 (VNII), 102-14 (VNII/VNB), MMRL2445 (VNII/VNB), 32-15 (VNII/VNB), 92-21 (VNI), and UGB047 (VNI). FACS profiles of the haploid control strain JEC21, isolates UGB047 (VNI), MAS93-0102 (VNII), 102-14 (VNII/VNB), Bt158 (VNB), and MMRL1351 (VNIII) are shown on the right. 1n, 2n, and 4n indicate nuclear DNA content. The *x* axis indicates fluorescence intensity reflecting the DNA content and the *y* axis indicates cell count.

Four hundred and eighty nine recorded *C. neoformans* clinical and environmental isolates collected from six continents (Asia, Africa, Australia, Europe, and North and South Americas) were tested for ploidy by fluorescence flow cytometry (FACS) (Table S1). Compared to the haploid control strain JEC21, 38 (7.8%) of these isolates were found to contain twice the cellular DNA content based on FACS analysis and were therefore diploid (FACS profiles of a few selected isolates are shown in Figure 1). These strains were checked for nuclear straining by fluorescence microscopy prior to FACS analyses. They were all uninucleate yeast cells, indicating that they are diploid and not dikaryons harboring two unfused haploid nuclei. Six additional isolates are likely to be aneuploid between haploid and diploid based on their FACS profile and may represent degenerate diploids. No ploidy higher than diploid, such as triploid or tetraploid, was observed (Table S1). Together with the observation that some of the diploid strains could become aneuploid or haploid (Table 1) through unknown mechanisms (chromosome loss or mitotic gene conversion), our results suggest that higher ploidy might be unstable. There was no association of diploidy with any geographic region, indicating that the global *C. neoformans* population is a mixture of haploids and diploids, and diploids represent ∼8% of the total population.

**Table 1:** Genotype of diploid strains.

Strain	Molecular Type		Location	Agglutination assay^1^	Serotype 2	*MAT* 2	Genotype
	AFLP(Eac)	AFLP(Etg)					
92-21	*VN I*	*VN I*	USA	*A*	*A*	*α*	*αAAα*
C24	*VN I*	*VN I*	Brazil	*A*	*A*	*α*	*αAAα*
SB4	*VN I*	*VN I*	Brazil	*A*	*A*	*α*	*αAAα*
UGB047	*VN I*	*VN I*	Uganda	*A*	*A*	*α*	*αAAα*
20020.092	*VN I*	*VN I*	Uganda	*A*	*A*	*α*	*αAAα*
20021.095	*VN I*	*VN I*	Uganda	*A*	*A*	*α*	*αAAα*
MMRL743	*VN I*	*VN I*	Italy	*A*	*A*	*α*	*αAAα*
MMRL752	*VN III*	*VN III*	Italy	*D*	*A*	***a***	***a*** *ADα* 6
MMRL795	*VN I*	*VN I*	Brazil	*A*	*A*	*α*	*αAAα*
MMRL799	*VN I*	*VN I*	Brazil	*A*	*A*	*α*	*αAAα*
MMRL1088 *	*VN I*	*N/A*	Japan	*A*	*A*	*α*	*αAAα*
MMRL1368	*VN I*	*VN I*	Argentina	*A*	*A*	*α*	*αAAα*
MMRL1370	*VN I*	*VN I*	Argentina	*A*	*A*	*α*	*αAAα*
MMRL2445 * 3	*Hybrid*	*Hybrid*	Brazil	*A*	*A*	*α*	*αAAα*
MMRL1351	*VN III*	*VN III*	France	*D*	*D*	***a***	*-AD* ***a*** 7
VPCI 73	*VN I*	*VN I*	India	*A*	*A*	*α*	*αAAα*
8F.12.8.02	VN I	VN I	Tanzania	A	*A*	*α*	αAAα
49F.11.97	*Hybrid*	*Hybrid*	Tanzania	A	*A*	*α*	αAAα
MAS92-0333	*N/A*	*VN─*	USA	*A*	*A*	*α*	αAAα
MAS93-0102	*VN─*	*VN─*	USA	*A*	*A*	*α*	αAAα
32-14	*N/A*	*Hybrid*	USA	*A*	*A*	*α*	αAAα
32-15	*VN─*	*Hybrid*	USA	*A*	*A*	*α*	αAAα
102-14	*VN─*	*Hybrid*	USA	*A*	*A*	*α*	αAAα
Bt2 4	VN I	VN I	Botswana	A	A	α	αAAα
Bt9 4	VN I	VN I	Botswana	A	A	α	αAAα
Bt29 4	VN I	VN I	Botswana	A	A	α	αAAα
Bt30 4	VN I	VN I	Botswana	A	A	α	αAAα
Bt50 4	VN B	VN B	Botswana	A	A	α	αAAα
Bt100 4	VN B	VN B	Botswana	A	A	α	αAAα
Bt107 4	VN B	VN B	Botswana	A	A	α	αAAα
Bt114 4	VN I	VN I	Botswana	A	A	α	αAAα
Bt153 4	VN I	VN I	Botswana	A	A	α	αAAα
Bt158 4	VN B	VN B	Botswana	A	A	α	αAAα
Bt163 4	VN I	VN I	Botswana	A	A	α	αAAα
1033 5	VN II	VN II	Australia	A	A	α	αAAα
1035 5	VN II	VN II	Australia	A	A	α	αAAα
1052 5	VN II	VN II	Australia	A	A	α	αAAα
1054 5	VN II	VN II	Australia	A	A	α	αAAα
KN#1	VN IV	VN IV	Lab	*D*	*D*	***a***	***a*** *DD* ***a***
KN#24	VN IV	VN IV	Lab	*D*	*D*	***a***	***a*** *DD* ***a***
KN4B4#10 *	VN I	VN I	Lab	*A*	*A*	*α*	*αAAα*
KN5B4#1	*VN I*	*VN I*	Lab	*A*	*A*	***a*** */α*	***a*** *AAα*
KN2B5#17	VN I	VN I	Lab	*A*	*A*	***a***	***a*** *AA* ***a***
KN2B5#18 *	VN I	VN I	Lab	*A*	*A*	*α*	*αAAα*
KN2B5#19	VN I	VN I	Lab	*A*	*A*	***a*** */α*	***a*** *AAα*
KN4B7#5	VN I	VN I	Lab	*A*	*A*	***a*** */α*	***a*** *AAα*
KN4B7#16	VN I	VN I	Lab	*A*	*A*	***a***	***a*** *AA* ***a***

Texts in italics indicated that information contained was generated for this study, and the rest was compiled from other sources.

“VN─” indicates that the AFLP method used here cannot distinguish the molecular types VNB and VNII.

“KN”- all KN strains were generated from the **a**-α crosses originally designed to obtain congenic pairs for the serotype D strain NIH433 and the serotype A strain H99 [31],[70].

1 based on Crypto Check agglutination assay.

2 Based on serotype and mating type specific PCR of *SXI1α/2* ***a*** and *STE20α/* ***a***.

3 The MMRL2445 isolate is likely of VNB molecular type based on MLST analysis. The AFLP is probably not discriminatory for this isolate.

4 Genotype information about Bt strains was obtained from previous reports [40],[120].

5 Genotype information about Australian strains was obtained from a previous report [43].

6 See a previous report [46].

7 based on serotype- and mating-type- specific PCR of *SXI1/2* and *STE20* where only D**a** alleles were amplified. Serotype-specific PCR of *GPA1* and *PAK1* indicated that both serotype A and D alleles were present.

*: strains with unstable diploidy. The mechanisms of the diploidy instability in these strains are not clear. However, based on recent comparative genomic studies in *C. neoformans* and *Candida albicans* [123]–[125], it is possible that the population might have cells of different ploidy due to chromosomal loss or mitotic conversion.

### A majority of diploid *C. neoformans* isolates are serotype A isolates

Because most diploids previously reported are inter-varietal AD hybrids, the genotype of the natural diploids identified by FACS analysis was examined by slide agglutination assay with the Crypto Check kit (Iatron Labs., Tokyo, Japan) according to the manufacturer's instruction [61]. The majority of the natural diploids tested (19/21) typed as serotype A and two typed as serotype D. Because serotyping by this agglutination test is based on the affinity binding of adsorbed antibodies for capsular epitopes, the specificity of these reactions could vary. Furthermore, this serotyping kit is no longer commercially available. Therefore, the serotype of these natural diploid isolates was further examined by analyzing their AFLP patterns using two different sets of primers (EAC and ETG) as previously described [40],[46],[62]. The AFLP analysis method used here can distinguish different serotypes but does not have the resolution to resolve molecular types within the serotype A isolates. As shown in Figure 1 and summarized in Table 1, four different AFLP banding patterns were observed representing different genotypic subpopulations: VNI (serotype A), VN─ (VNII or VNB, serotype A), VNIII (serotype AD hybrid), and a hybrid pattern representing a composite between molecular types within serotype A (AFLP patterns with the ETG primer set for a few selected isolates are shown in Figure 1). Multi-locus sequence typing (MLST) analyses indicate that these hybrids are VNII/VNB (see the section about phylogenetic analysis for details). The two diploids (MMRL752 and MMRL1351) that were typed as serotype D by the agglutination assay were found to be AD hybrids displaying a VNIII pattern (Table 1, Figure 1). Four strains displayed a unique intra-varietal hybrid pattern. Intra-varietal serotype A hybrids between VNII and VNB have recently been reported [47] These observations suggest that intra-varietal hybridization events are likely to be common in nature. These hybrids may not be easily recognized due to the insensitivity of current typing techniques applied in many ecology and epidemiology studies.

The finding that the majority of the diploids from this sample set are serotype A isolates is consistent with the fact that serotype A is the most common serotype and is responsible for 95% of human infections worldwide [14],[63]. This finding further indicates that diploids occur naturally as both inter-varietal (AD and BD hybrids [64]) and intra-varietal (A^1^A^2^ hybrids) forms.

### All natural serotype A diploids are α mating type

To further characterize the genotype of these diploid isolates, PCR amplification of the serotype- and mating-type-specific genes *SXI1α*/*2* ***a*** and *STE20α*/***a*** located within the mating type locus (*MAT*) was used to molecularly define their serotype and mating type [22],[46]. The *SXI1α*/*2* ***a*** and *STE20α*/***a*** genes are physically distant from each other within the mating type locus that spans over 100 kb [65], and it would be rare to have mutations or rearrangements affecting both genes. In addition, Ste20 plays important roles in growth and mating in serotype A strains [66] and natural mutations such as indels, which would most likely lead to failure of the PCR screening approach, are unlikely to occur. Genotyping a large number of natural strains in previous studies also found no indels in the *SXI1*α serotype A specific gene [41],[46]. Thus, the serotype A alleles of both *SXI1α*/*2* ***a*** and *STE20* are likely to be intact in natural populations. PCR analysis of every diploid strain using each serotype- and mating-type-specific primer set was repeated at least twice and was performed with all the positive and negative controls (Aα, A**a**, Dα, D**a**).

All of the natural serotype A diploid isolates were positive for serotype A mating type α specific amplicons but negative for mating type **a** specific amplicons, based on this PCR analysis (Table 1). Thus, these diploids possess only α mating type information. This result is consistent with the predominance of the α mating type in the *C. neoformans* population, particularly among strains of serotype A. It is interesting to note that all of the Botswanan diploids identified were also of α mating type (Table 1), even though 10% of the Botswanan strains are **a** mating type [40]. One AD hybrid (strain MMRL752) contained **a** alleles from serotype A and α alleles from serotype D and is apparently derived from mating between A**a** and Dα cells (Table 1). The other AD hybrid (strain MMRL1351) contains both serotype A and D alleles of the *GPA1* and *PAK1* loci located in other genomic regions, but this strain is only positive for D**a** amplicons for the mating type locus based on our PCR analyses. The mechanisms that produced this isolate likely involve an ancestral αAD**a** hybrid with a secondary loss of the Aα mating type locus allele (−AD**a**).

### Diploid α isolates contain two copies of α mating type genes

The natural diploid serotype A isolates with only the α mating type locus based on PCR analysis could: 1) contain two *MATα* alleles, 2) only one mating type α allele as an aneuploid (2n−1), or 3) a cryptic **a**/α heterozygous *MAT* locus that eluded detection by this PCR method. Because the mating type locus of *C. neoformans* contains more than 20 genes and spans over 100 kb [65], it is one of the most complex mating type loci in the fungal kingdom. To determine the genetic composition of the *MAT* locus of the serotype A α diploids, genes within the *MAT* locus of selected diploid isolates were examined by comparative genome hybridization (CGH).

**Figure 2:**
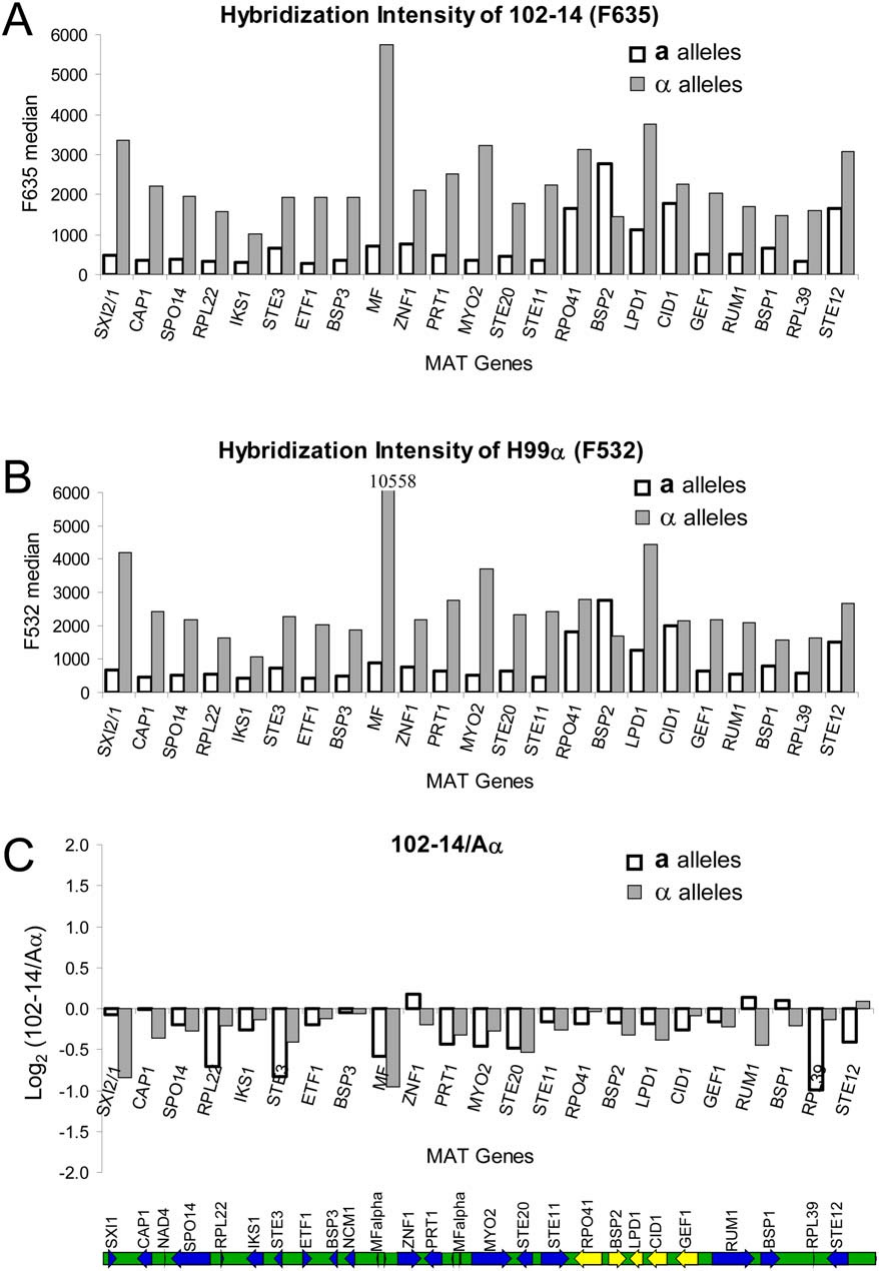
Comparative genome hybridization of the diploid isolate 102-14 mating type locus. Genomic DNA from the environmental isolate 102-14 and control strain H99 (Aα) were fragmented, labeled with fluorescent dyes, and competitively hybridized to a 70-mer genomic microarray. The fluorescent signal intensity was normalized across the genome. The average fluorescent intensity of three independent replicates of serotype A specific genes in the mating type locus for the sample 102-14 (red channel F635) and for the Aα haploid control strain H99 (green channel F532) are shown in panels A and B, respectively. The log_2_ values of the fluorescence intensity ratios between 102-14/H99 are shown in panel C. White bars indicate a specific alleles and grey bars indicate α specific alleles. A schematic representation of the serotype A mating type locus is illustrated at the bottom [65]. Blue color indicates intergenic regions and yellow color indicates highly conserved genes.

Genomic DNA of four selected natural diploids of different molecular types [102-14 (VNB/VNII), MMRL2445 (VNB/VNII), MMRL799 (VNI/VNI), 1033 (VNII/VNII), and the haploid control H99 (Aα, VNI)] were labeled with fluorescent dyes and competitively hybridized to a genomic microarray slide containing mating-type and serotype-specific 70-mers corresponding to each **a** and α *MAT* gene allele from all 4 serotypes. The arrays were all normalized. The control and the samples displayed similar hybridization patterns in genomic regions other than the mating type locus (data not shown). To determine whether diploid strains have an α mating type locus or harbor a cryptic *MAT* **a**/α heterozygous locus, the hybridization signals for *MAT*α and *MAT* **a** alleles were analyzed. As expected for α strains, the diploid isolate 102-14 hybridized strongly to all α specific probes whereas it hybridized weakly to most **a** specific probes (Figure 2A). Strong hybridization signals were observed for both α and **a** probes for several genes (*RPO41*, *BSP2*, *LPD1*, *CID1*, and *STE12*). The first four of these genes are highly conserved and were probably acquired most recently into the mating type locus [65] (Figure 2, bottom). Thus cross-hybridization between **a** and α alleles is likely to occur, resulting in strong hybridization signals for both the **a** and α alleles. Cross-hybridization between **a** and α alleles for *STE12* could be due to non-specificity of the designed 70 mer. To ensure that this hybridization pattern was interpreted correctly, the hybridization pattern for the control Aα H99 strain was also analyzed. As shown in Figure 2B, the α control strain displayed a similar hybridization profile supporting the conclusion that the diploid isolate 102-14 is indeed an α isolate. Similar patterns were also observed for the three other diploid isolates analyzed (data not shown). The hybridization patterns at the mating type locus from α cells observed in this study are opposite from that of **a** cells, whose genomic DNA hybridizes strongly to **a** alleles but weakly to α alleles on the same array [67].

Next, the copy number of the α mating type alleles in these diploids was determined to be two. As a diploid, 102-14 has twice the genomic DNA content per cell compared to the haploid control H99 Aα strain. Because an equal amount of DNA from the diploid sample and the haploid control were used, normalization of the whole genome array across the entire genome other than *MAT* artificially rendered the genomic DNA content between 102-14 and H99 equivalent, yielding a 0 in the Log_2_ ratio between the sample and the control (fold ratio = 1). In other words, the 2n ploidy of 102-14 isolate was treated as 1n in this analysis. After normalization, the Log_2_ ratio of fluorescence intensity was calculated between the diploid isolate 102-14 and the control haploid Aα H99 for all serotype A *MAT* **a** genes and *MAT*α genes. If 102-14 had only one mating type α allele as an aneuploid (2n−1, or 1n−½ with normalization), the fold difference between 102-14 and control H99 for the mating type genes should have been ½, giving a Log_2_ ratio of −1. If 102-14 has two mating type α alleles (2n, or 1n with normalization), the fold difference between the sample and control for the mating type genes should be 1, yielding 0 to the Log_2_ ratio between 102-14 and H99. Most of the values were found to be close to 0 (most Log_2_ ratio of fluorescence intensity was within −0.5∼0.0, meaning that the fold difference between the sample and control fell into the range of 0.7∼1) (Figure 2C). This means that the fold difference between the control and the sample in the mating type region is similar to other genomic regions. Thus, this observation indicates that 102-14 is not an aneuploid at the mating type locus and indeed has two α alleles at the mating type locus. Because there were only α genes and no **a** genes in the control, and the hybridization pattern of the sample 102-14 was similar to the Aα H99 control, this again confirms that the sample diploid is an α strain. The Log_2_ (102-14/Aα) values of most α genes were between −0.5 and 0, instead of centered around 0, as would be expected from a fold difference ratio = 1.0. This minor hybridization difference is most likely attributable to better hybridization of the control H99 to this array since the mating-type- and serotype-specific 70-mer nucleotide probes on the array were designed from the sequence of H99 (Griffith et al., unpublished results). Similar patterns were observed for the other three isolates MMRL2445, MMRL799, and 1033 (Figure S1).

**Figure 3:**
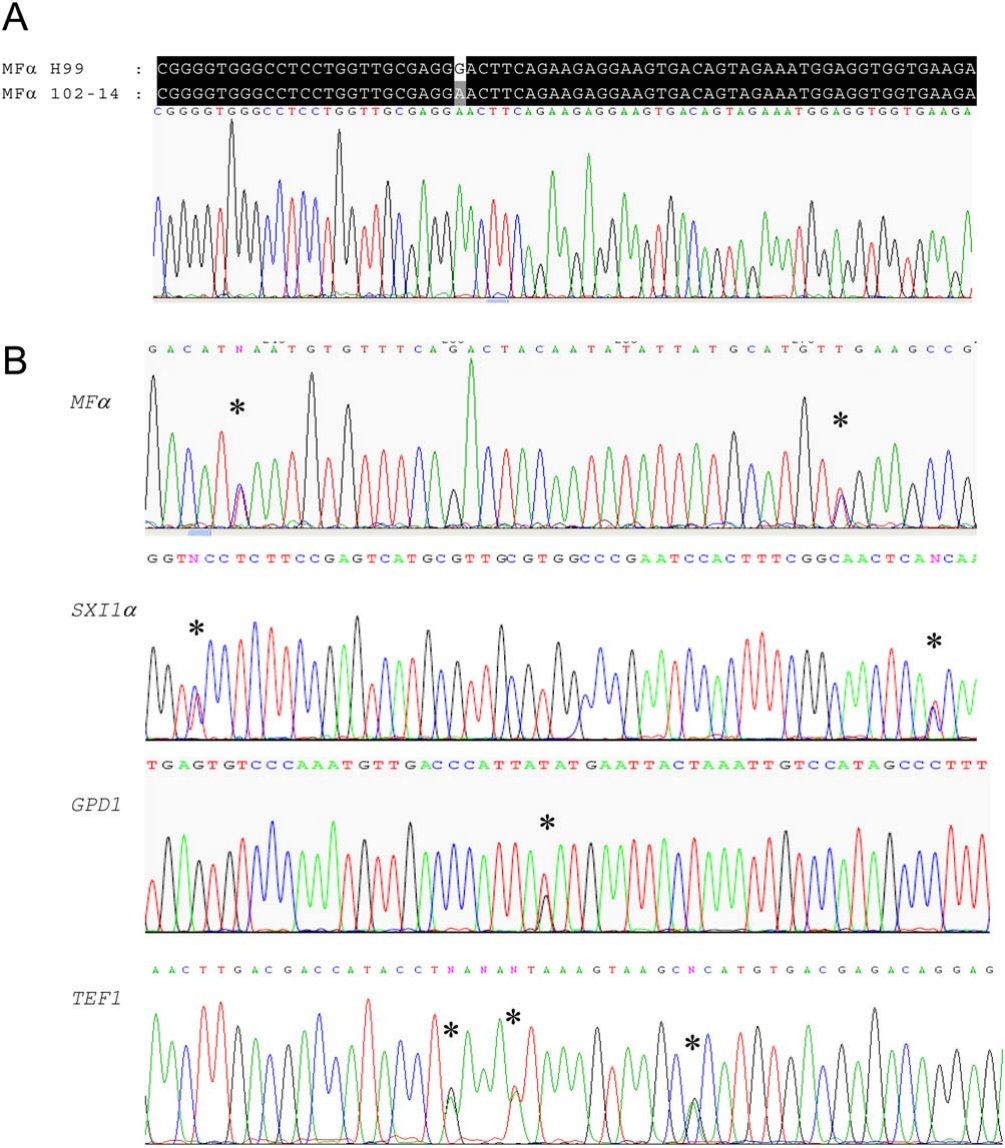
Nucleotide polymorphisms in the serotype A intra-varietal hybrids. A.) The *MFα1* pheromone gene of isolate 102-14 differs from that of H99 reference strain in the region covered by the 70 mer on the microarray. B.) Polymorphisms in a portion of the *MFα1*, *SXI1α*, *GPD1*, and *TEF1* genes are shown for strain 102-14. Stars indicate the polymorphic sites.

The hybridization signals for the *MFα* pheromone gene were strongest for both the sample diploid 102-14 and the control haploid H99 (Figure 2) due to the presence of multiple pheromone gene copies in the mating type locus (4 copies for H99). However, the hybridization signal for 102-14 was only half of the control, yielding a log_2_ ratio 102-14/H99 close to −1 (Figure 2C). This could be due to the presence of fewer *MFα* gene copies in isolate 102-14 or reduced hybridization of the *MFα* genes from isolate 102-14. As mentioned previously, because the mating-type- and serotype-specific 70-mer oligonucleotide probes were designed from the H99 sequence, weaker hybridization signals are expected for the other strains, especially for those with more divergent genes. Sequencing the *MFα* pheromone region covered by the 70 mer from isolate 102-14 revealed a nucleotide difference from that of H99 occurring at position 26 (Figure 3A), supporting the interpretation of weaker hybridization for isolate 102-14.

The CGH analyses indicate that the diploid strains contain two α alleles at the mating type locus. However, because the array used was not a tiling array, and there is only one 70-mer probe for each gene, whether their mating type locus is completely intact or bears alterations or rearrangements in regions not covered by the probes can not be detected at this level of resolution.

### Diploidization via same-sex mating mechanisms occurs in nature

**Figure 4:**
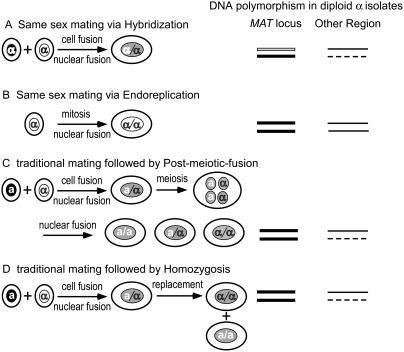
Mechanisms for the generation of diploid α isolates. A) Mating between two genetically distinct cells of the same mating type, most commonly α, leads to a cell fusion event followed by a nuclear fusion event that produces a diploid cell heterozygous at both the *MAT* locus and other genomic regions. B) Fusion between two isogenic α cells or endoreplication of a single cell nucleus without cytokinesis (cell separation) both produce diploid cells that are identical at the *MAT* locus and other genomic regions. C) Post meiotic fusion after traditional mating between α and a cells generates α/α, a/α, and a/a diploid cells. Inner circles represent nuclei and outer circles represent cells. D.) Homozygosis of the mating type locus following traditional mating between cells of opposite mating types, α and a, leads to production of diploid α/α and a/a diploid cells. Homozygosis can occur either due to mitotic recombination or chromosomal loss and duplication of the retained homolog. The resulting diploid α cell is thus homozygous at the *MAT* locus but heterozygous at other genomic regions. α or a indicates the mating type of the cell. Lines on the right represent genomic loci with solid, open, or dashes designating different alleles.

α/α diploids could arise via several alternative mechanisms. They could be derived from either α-α same-sex mating or **a**-α classical mating via four alternative models (Figure 4). In model 1, during same-sex mating, an α cell fuses with another α cell that is genetically distinct (Figure 4A, hybridization) or identical (Figure 4B) producing a heterozygous or homozygous diploid cell, respectively. In model 2, a mitotic event without cytokinesis gives rise to homozygous diploid α/α cells (Figure 4B, Endoreplication). In model 3, during traditional **a**-α mating, a heterozygous **a**/α diploid cell is normally produced. The resulting **a**/α diploid state is usually transient and undergoes meiosis immediately, generating four haploid nuclei. In rare cases, these meiotic haploid nuclei may randomly fuse to produce α/α, **a**/α, and **a**/**a** diploid cells in a ratio of 1∶ 2∶ 1 (post-meiotic fusion) [68] (Figure 4C). Finally, in model 4, under certain conditions, such as high temperature, the diploid **a**/α state is stabilized and cells are grown in the yeast form [69]. It is possible that the mating type locus of diploid **a**/α cells undergoes loss of one of the mating type alleles with subsequent duplication of the remaining allele, producing diploid α/α and **a**/**a** diploid cells (homozygosis) (Figure 4D).

To determine by which mechanisms α/α diploids arose, polymorphisms in genetic loci in the mating type locus (*SXI1α*, *STE20α*) and other genomic regions (*IGS1*, *PLB1*, *TEF1*, and *GPD*1) were examined. If a diploid α/α isolate resulted from a hybridization event, the strain would show nucleotide polymorphisms at both the *MAT* locus and other genomic regions (model 1, Figure 4A). If the diploid α/α isolate was derived from endoreplication or fusion between two isogenic α cells, these loci would have identical nucleotide sequences (model 1B or 2, Figure 4B). If the α/α diploid was derived by homozygosis of the mating type locus or by post-meiotic-fusion following traditional **a**-α mating, it would be monomorphic at the *MAT* locus, but polymorphic at other genomic loci (models 3 and 4, Figure 4C and 4D).

**Figure 5:**
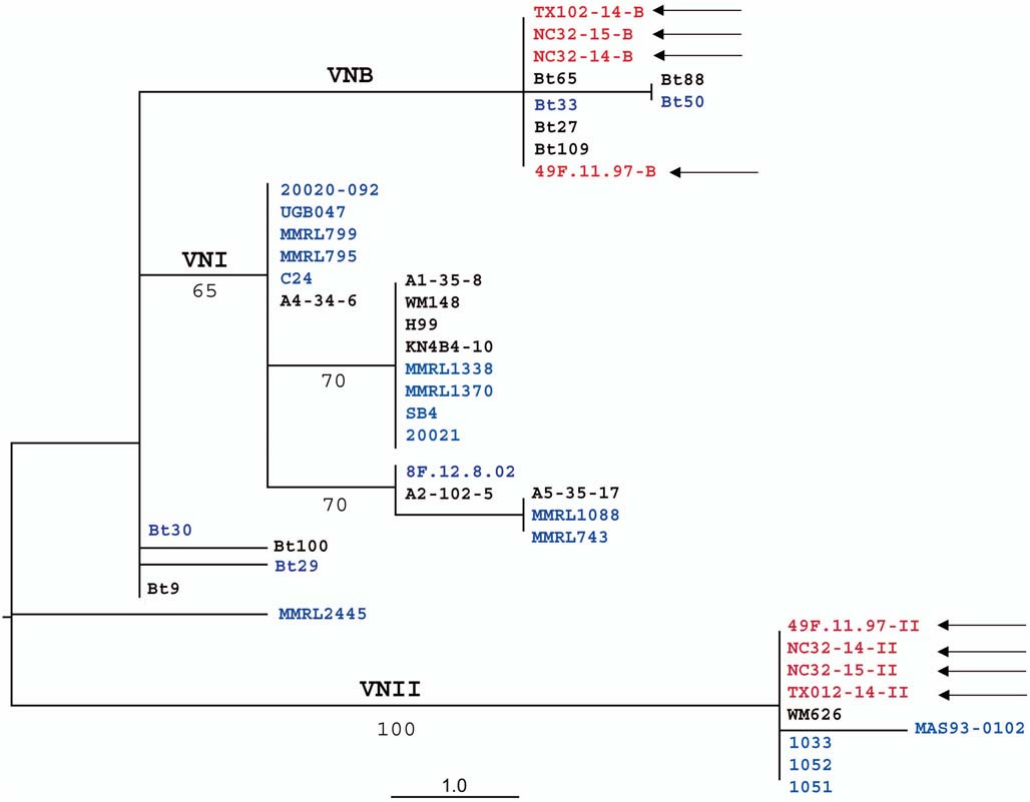
Single maximum parsimony tree inferred from the combined gene genealogies of *GPD1* and *PLB1*loci. Diploid isolates from this study are shown in blue, cloned alleles from the VNII/VNB hybrids are shown in red and marked with arrows, haploid typing strains are black. Three molecular types are indicated, e.g. VNI, VNII, and VNB. Bootstrap values of major branches are indicated under the lines.

**Figure 6:**
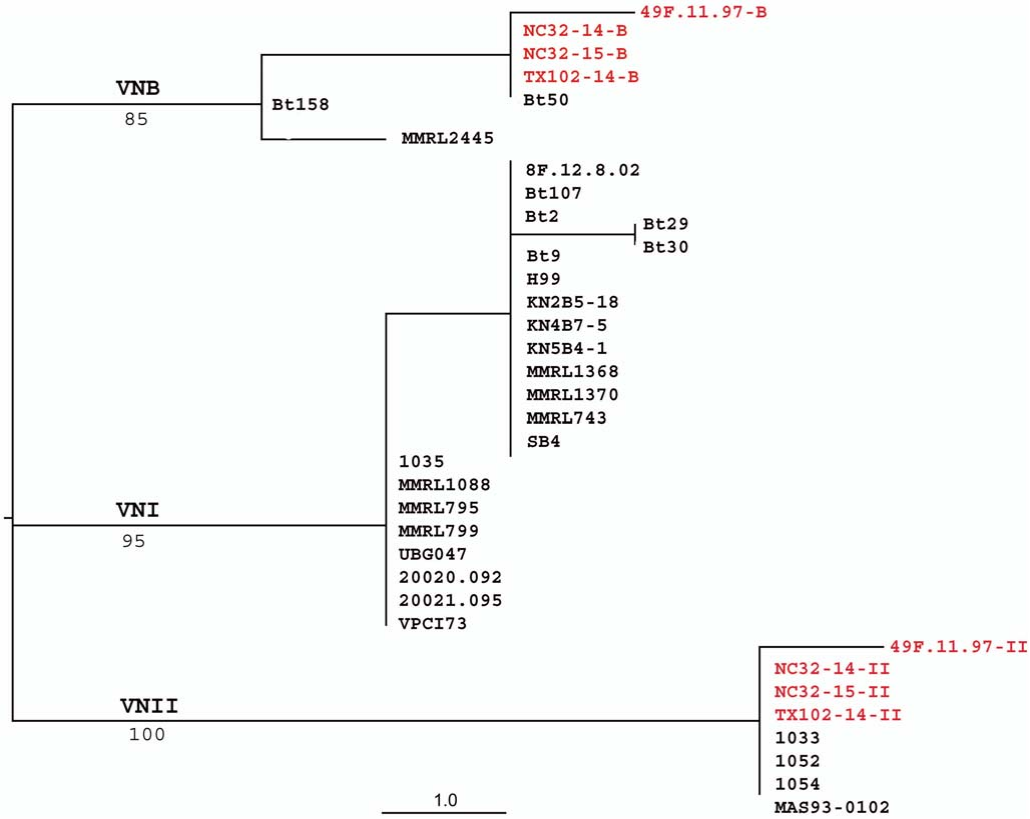
Single maximum parsimony tree inferred from the *STE20*. Cloned alleles from the VNII/VNB hybrids are shown in red. Bootstrap values of major branches are indicated under the lines.

Analysis of strains 49F.11.97, 32-14, 32-15 and 102-14 revealed no polymorphism in the *IGS1* locus. All four strains contained a single *IGS1* allele, which clustered with the *IGS1* sequences from the VNII molecular type (Figure S2). However, polymorphisms were present in both the mating type (*SXI1α* and *STE20α*) and other genomic loci (*GPD1*, *PLB1*, *TEF1*) (Figure 3 and data not shown). The 102-14 *MFα* gene in regions outside of the region covered by the 70 mer oligo array also showed polymorphisms (Figure 3). Subsequent cloning of two different alleles for the *STE20* gene within the mating type locus and *GPD1* and *PLB1* in other genomic regions (see Figure 5 and Figure 6) indicates that these diploid α/α strains were indeed derived from same-sex mating through fusion between two genetically distinct α cells within the same serotype, similar to recently identified inter-varietal αADα strains [46]. These findings are consistent with AFLP genotyping that indicated that these strains are serotype AA hybrids (Table 1). In the other natural α/α diploids, including diploids isolated from Botswana where **a** cells are commonly present, no polymorphisms were observed in the sequenced fragments at the *MAT* locus or other locations. Consequently, these diploids probably originated by fusion between two isogenic α cells or by endoreplication. These observations support the view that autopolyploids are more common than allopolyploids because clone mates are more often in close proximity and mutually compatible.

### Diploidization via conventional mating mechanisms occurs under laboratory conditions

The analyses of nucleotide polymorphisms did not detect natural α/α diploid strains that derived from conventional **a**-α mating. This is likely due to the infrequency of natural **a**-α mating given the rare presence of **a** isolates in the environment. Furthermore, *MAT* homozygosis or post-meiotic-fusion events are likely to be rare, anomalous events during **a**-α mating, further reducing the frequency of occurrence.

To examine whether diploid α/α strains can be generated by **a**-α mating, 201 laboratory strains were tested for ploidy by FACS analysis. These strains were derived from **a**-α crosses during construction of congenic strain pairs for the serotype D strain NIH433 and the serotype A strain H99 [31],[70] (Table S1). Nine strains were found to be diploid. The configuration of their mating type locus was determined by PCR amplification of the serotype- and mating-type-specific *SXI1α/2* ***a*** and *STE20α/* ***a*** genes [46]. As shown in Table 1, diploid strains with a single mating type of either **a** or α alleles were identified, as well as diploids with an **a**/α heterozygous mating type locus. Similar to the natural diploids described above, these laboratory isolated diploids likely contain two copies of *MAT*. The lab diploid strain KN4B7#16 was confirmed to contain two *MAT* **a** alleles in another study [67]. Because diploids of all three types of mating type composition were identified (α/α, **a**/α, and **a**/**a**), they were likely products of post-meiotic fusion events, although the probability that they originated by traditional mating followed by homozygosis cannot be excluded (Figure 4C, D).

Post-meiotic random fusion of two of the four recombinant nuclei has been proposed to explain the origins of AD hybrids homozygous at the mating type locus [68]. Based on our observations, this process could conceivably occur under laboratory conditions. However, it is doubtful that post-meiotic nuclear fusion generated most of the AD hybrids in nature because diploid strains with α/α, **a**/**a**, or **a**/α mating type composition would be predicted to be produced in a 1∶1∶2 ratio (see Figure 4C). This is not the case as most natural AD hybrids are either αAD**a** or **a**ADα, and only a minority of AD hybrids are αADα [46]. The existence of **a**AD**a** hybrids has not been reported. As described above, we have shown that diploid isolates of the same mating type can be generated in the laboratory by post-meiotic fusion or homozygosis events, but in natural settings, this mechanism is unlikely to be a common mechanism of diploidization.

### Phylogenetic analysis indicates that the diploids are genetically distinct isolates

To examine whether these diploids were derived independently, or rather produced by clonal expansion of a single ancestral diploid, genetic diversity of these isolates was analyzed by comparing their nucleotide sequences at several genetic loci including the *IGS1*, *GPD1*, *PLB1*, and *STE20α* loci. Combined *PLB1+GPD1* phylogeny is shown in Figure 5, and a phylogenetic tree based on *STE20α* is shown in Figure 6. The phylogenetic trees illustrate that diploid strains are found among of all three molecular types of serotype A: VNI, VNII, and VNB. Phylogenetic analysis of *PLB1*, *GPD1*, *STE20*, and *IGS1* loci, identified 10 different genotypes among the diploid strains of serotype A, suggesting that diploid strains have originated on multiple independent occasions. The two allele sequences of *PLB1*, *GPD1* and *STE20* loci of the diploid hybrids (102-14, 32-14, 32-15, and 49F-B) were obtained via PCR-amplification followed by TA cloning into *E. coli*, and subsequent sequencing from individual plasmids. As shown in Figure 6, all of the four hybrid strains harbor two alleles, one clustered with molecular type VNB and the other with VNII, indicating that these hybrids are intra-varietal VNII/VNB hybrids. The fact that these hybrids harbor two α alleles of *STE20* (one VNB type and one VNII type) further supports that these hybrids are derived from α-α unisexual mating.

### Diploid isolates vary morphologically and phenotypically

**Figure 7:**
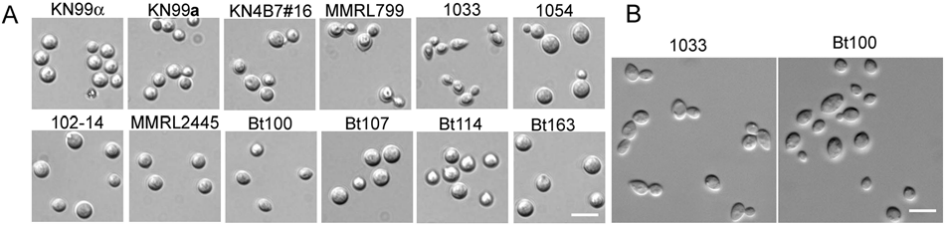
Morphology of diploid yeast cells varies. Haploid isolates KN99α and KN99a, and diploid isolates KN4B7#16, MMRL799, 1033, 1054, 102-14, MMRL2445, Bt100, Bt107, Bt114, and Bt163 were grown in YPD medium at 30°C overnight, washed three times with water, and examined microscopically (A). A larger field is shown for strains 1033 and Bt100 in a second experiment (B). Scale bar, 10 µm.

The morphology of selected diploid isolates was further examined microscopically. The yeast cells of the majority of the diploid isolates were typically spherical, similar to the well-studied congenic laboratory reference strains H99, KN99α, and KN99**a** (Figure 7). The cell shape of these strains was not affected by growth in rich or synthetic liquid media (YPD or YNB, respectively) or by temperature (30°C or 37°C) (data not shown). However, cells of two diploid isolates, 1033 and Bt100, were elliptical, and this morphology did not change when the strains were cultured in YPD or YNB at 30°C or 37°C. Whether variation in cell shape has any significance in survival in the environment or host is not known. The majority of the diploid isolates appeared larger in size than the haploid controls (Figure 7). An association between larger cell size and higher ploidy has been reported in other fungi [12],[71],[72], as well as in *C. neoformans* [67],[73].

As an environmental pathogen, *C. neoformans* does not require an animal host to complete its life cycle. However, several well-studied traits are considered important for both its survival in the environment and its pathogenesis in mammalian hosts. These properties include melanization, capsule production, the ability to grow at extreme temperatures, and resistance to UV irradiation [19],[60],[74]. Recently, *C. neoformans* has also been shown to undergo invasive growth under conditions of limited nitrogen [75]. Consequently, the following phenotypes were evaluated in selected diploids: capsule production, growth at 4°C and 37°C, melanization, sensitivity to UV irradiation, and invasive growth.

**Figure 8:**
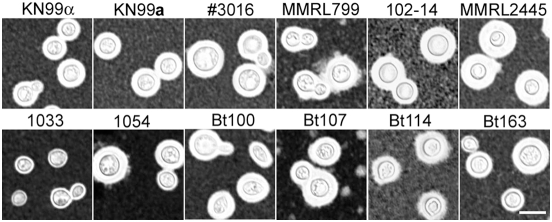
Natural diploid isolates produce capsule. Haploid isolates KN99α and KN99a, and diploid isolates KN4B7#16, MMRL799, 102-14, MMRL2445, 1033, 1054, Bt100, Bt107, Bt114, and Bt163 were grown in YPD medium at 30°C overnight, washed three times with water, and then incubated on DME solid medium at 37°C for 48 hours. Cells were then scraped from the plate, suspended in India ink, and observed microscopically for capsule production. A white halo surrounds the yeast cells when the ink is excluded by the capsule. Scale bar, 10 µm.

The cryptococcal polysaccharide capsule protects *Cryptococcus* cells from dehydration in the environment and inhibits host defenses [76]–[79]. All strains tested were able to produce capsule after incubation at 37°C in DME medium, as evidenced by a white halo surrounding the yeast cell in a suspension of India ink particles (Figure 8). Isolate 1033 produced smaller capsules compared to other isolates under these conditions. Capsule size and cell morphology were not correlated, as the other oval shaped isolate Bt100 produced larger capsules under the same conditions (Figure 8).

**Figure 9:**
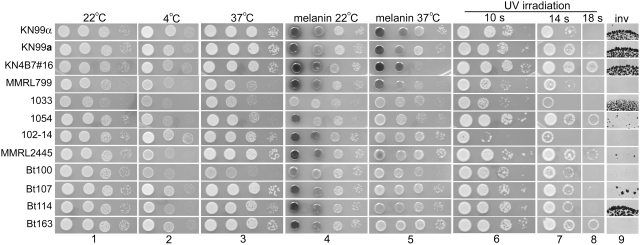
Diploid isolates exhibited phenotypic variation. Haploid isolates KN99α and KN99a, and diploid isolates KN4B7#16, MMRL799, 1033, 1054, 102-14, MMRL2445, Bt100, Bt107, Bt114, and Bt163 were serially diluted (10×) and grown on YPD medium at 22°C for two days as controls for growth (column 1). Cells were grown on YPD medium at 4°C (10 days, column 2) and 39°C (2 days, column 3) to assay growth at extreme temperatures. Cells were grown on Rose Bengal medium at 22°C (column 4) and 37°C (column 5) for two days to assay melanization. Cells were spotted on YPD medium and subjected to UV irradiation for 10, 14, 18 sec (∼48 mJ/cm^2^) and then incubated at 22°C for three days to assay resistance to UV irradiation (columns 6–8). Cells were grown on SLAD medium at 30°C (last column) for five days to assay invasive growth.

The ability of *C. neoformans* to propagate in a broad range of temperatures enhances its survival in environments such as sub-Saharan Africa, where daily or seasonal temperatures fluctuate widely. Growth of selected diploids and haploid controls at 4°C was examined and variation in growth rate at this low temperature was observed (Figure 9, column 2). Diploid isolate 102-14 and two Botswanan strains (Bt107 and Bt163) grew well at 4°C, similar to the lab reference strains. But two other diploid isolates 1033 and Bt100, grew poorly at this low temperature. The ability to grow at 37°C is essential for mammalian pathogens, which sets most pathogenic fungi apart from non-pathogens. All of the diploid strains tested were able to grow at 37°C, although their rate of growth varied (Figure 9, column 3). There appears to be an association between growth at low and high temperatures as isolates that grew well at high temperature also grew well at low temperature. The only exception was isolate MMRL2445, which grew poorly at 4°C but very well at 37°C.

*C. neoformans* can produce melanin by oxidizing a variety of diphenolic substrates, including the neurotransmitter L-dihydroxyphenylalanine (L-DOPA). Melanin is deposited in the cell wall and protects fungal cells from UV irradiation in the environment and toxic free-radicals generated by host defenses during infection [80],[81]. Variation in the rate of melanization is reflected in the pigmentation of the yeast colony. All of the isolates tested were capable of melanin production and their yeast colonies turned completely dark on Rose Bengal medium containing L-DOPA after incubation at 22°C for three days (data not shown). However, variation in the rate of melanization among the isolates was apparent at two days: isolates 1033 and Bt100 were less melanized (Figure 9, column 4). At 37°C, melanization was drastically reduced in the natural diploids and modestly reduced in the laboratory generated reference strains KN99α, KN99**a**, and the **a**/**a** diploid KN4B7#16 (Figure 9, column 5).

Resistance to UV irradiation also contributes to survival in adverse climates with high exposure to UV, such as the Kalahari Desert in Botswana [58]. It has been shown that higher ploidy confers resistance to UV irradiation [46]. The increased resistance of the laboratory generated **a**/**a** diploid KN4B7#16 to UV irradiation compared to its congenic haploid control strains KN99α or KN99**a** confirms a positive effect of higher ploidy (Figure 9, column 6). A range of sensitivity to UV irradiation was observed among the natural diploid isolates, and a few diploid isolates (1054, MMRL2445, and Bt163) were highly resistant. Because haploid progenitors of these natural diploids are unavailable, increased resistance to UV irradiation due to a ploidy effect is a likely explanation but cannot be definitely established.

Ploidy has no discernable effect on invasive growth because the **a**/**a** diploid strain KN4B7#16 was similarly invasive as its nearly isogenic haploid strain KN99**a** (congenic with KN99α/H99). Only two of the nine natural diploid strains tested grew invasively under nitrogen limiting conditions (Figure 9, column 9). Because *C. neoformans* is usually isolated from environments where nitrogen sources may not be limiting (avian guano, fruits, and decaying woods) [82]–[84], this observation suggests that the ability to undergo invasive growth may have evolved under unusual selective environmental conditions and thus this ability is not common to all *C. neoformans* isolates.

In conclusion, the diploid isolates vary in morphologies and phenotypes, which may reflect their genotypic differences and independent origins, consistent with our phylogenetic analyses.

## Discussion

A common feature of all organisms is that mutations are the ultimate means of generating the genotypic variation that enables a species to cope with environmental heterogeneity and stress. The effect of mutation on the diversity of a population is influenced by ploidy. Ploidy is an adaptive evolutionary trait and polyploidization is a driving force in the evolution of many eukaryotes [4], [85]–[89]. For example, a diploidization event followed by gene loss and accelerated evolution of one member of the retained duplicated genes gave rise to the extant lineages of *S. cerevisiae* [90]. In *S. cerevisiae*, ploidy regulates gene expression and adaptation in different environments [12],[52],[86],[91],[92], and differences in ploidy affect the paths for developing antifungal drug resistance [54]. Although haploid and diploid populations may be better at adapting to different environments, collectively, populations with both haploids and diploids may afford greater adaptability than those limited to only one or the other.

Most fungi exist predominantly in either a haploid or a diploid state. *C. neoformans* has been demonstrated to exist as a haploid, a diploid, or a dikaryon under laboratory conditions, but its ploidy distribution in natural populations has not been well studied. In this report, we found that close to 8% of the *C. neoformans* serotype A natural population are diploids, highlighting the potential significance of ploidy variation in this species. We did not find any natural triploids or tetraploids. This could be because higher ploidy in *C. neoformans* is not stable and/or cells of such higher ploidy are not evolutionarily fit. The observation that some diploid strains showed the tendency to reduce ploidy level to haploid, suggests that ploidy higher than haploid, the normal state of *C. neoformans*, might not be the most stable state for this fungus. Instability of higher ploidy has been demonstrated in other fungi such as *S. cerevisiae* and *C. albicans* where tetraploids and triploids shift their ploidy to the normal diploid state [85], [92]–[94].

The effects of ploidy shift and hybridization on the fitness and virulence in human pathogenic fungi are largely unexplored. Studies of strains in the H99 background demonstrate that higher ploidy confers increased resistance to UV irradiation but it exerts a modest detrimental effect on other phenotypes including melanization and virulence in mice [46],[67]. It would be interesting to determine if increased ploidy in other genetic backgrounds also exerts similar effects. Generating haploid isolates from these natural diploids would be necessary to address this question. Despite the fact that diploids might not always be more fit than haploids, there are potential benefits of having diploids in a haploid fungal species. First, polyploidy has protective effects and enables tolerance to genetic alterations. For example, the deleterious consequences of an extra chromosome can be less severe in diploid cells compared to haploids [95],[96]. Recessive mutations and deletions that are lethal in haploids are buffered in the diploid and polyploid state, resulting in subtle dosage effects. These effects could be potentially selected to generate adaptive variation. [97]. Second, shuttling between diploid and haploid states may enhance adaptation. As shown in the filamentous fungus *Aspergillus nidulans*, haploid strains generated from diploid strains via the parasexual cycle can reach the highest fitness under laboratory conditions, due to an inverse epistasis effect (antagonistic interactions between deleterious mutations) where mutations occurred in diploid nuclei that could be deleterious on their own in a haploid, but instead confer beneficial effects when combined [98]. The fact that about 0.01–0.1% *A. nidulans* are diploid may provide this species with just this type of advantage [99]. Third, diploid isolates can immediately produce resilient spores that can survive harsh environments and are easily dispersed, as diploids do not need to fuse with a partner to undergo sporulation. A similar scenario has been hypothesized for the existence of *S. cerevisiae* as a diploid [100]. Fourth, some of the *C. neoformans* diploids detected in this study could be nuclear fusion products of cells that were originally dikaryotic. *C. neoformans* can grow in a dikaryotic hypha form indefinitely if conditions permit and it is known that higher temperatures and other factors could stimulate dikaryoptic hyphae to become diploid yeast cells [69]. Being temporarily or for the longer term in a dikaryotic state (two separate nuclei in one cell) could offer unique opportunities for adaptation besides the effect of increased ploidy. For example, nuclear positioning in dikaryotic hyphae of the basidiomycetous fungus *Schizophyllum commune* affects gene expression pattern [101], and coevolution of the nuclei in one dikaryotic line could give rise to strains with better fitness [102]. Differences in gene regulation in a dikaryon versus a diploid have also been observed in the plant fungal pathogen *Ustilago maydis* [103]. Although it is not observed in *C. neoformans*, some other fungi can have variable numbers of nuclei per hyphal cell and unbalanced parental nuclei ratio, which provide additional means to generate genotypic and phenotypic variation [104].

Although cryptic autopolyploids are more common, many polyploidization events originate from hybridization between genetically distinct strains (allopolyploids). Hybridization provides an additional mechanism for generating genetic diversity as it results in a convergence of two different genomes and this can facilitate the exchange of adaptive alleles [4]. Hybridization can provide genetic variation within and between populations by yielding progeny more fit to novel or changing environments [105],[106]. Differential gene expression provides an additional source of genetic variation for the allopolyploids derived from hybridization [107]. However, there is limited information about hybridization and its associated ploidy shift in pathogenic fungal populations.

Most *Cryptococcus* hybrid strains characterized thus far are AD hybrids (hybrids between serotypes A and D). Hybrid vigor (heterosis) resulting from inter-varietal mating between serotype A and D has been implicated from ecological studies based on the appreciable frequency of AD hybrids in both clinical and environmental isolates [25], [73], [108]–[110]. Hybrid fitness has also been demonstrated for laboratory generated AD hybrids, including increased resistance to UV irradiation and significantly better growth at high temperature compared to isogenic diploids or their haploid parental strains [46],[67],[111]. While it is technically facile to detect AD hybrids through serological tests, detection of hybrids derived from cells of the same serotypes or of the same molecular type requires genotyping tests not commonly used in clinical labs. It is not surprising then that there has been no comprehensive documentation of intra-varietal hybrids. In this study, both inter-varietal and intra-varietal diploid hybrids of *C. neoformans* were identified. Subtle hybrid fitness may occur for intra-varietal hybrids such as those identified in this study and in another recent study [47]. Our observations suggest that this could be one of the mechanisms contributing to the genotypic and phenotypic diversity observed among *Cryptococcus* natural populations. The combined effect of increased ploidy and hybridization may contribute significantly to the development of a dynamic population that is well-suited in both the environment and the host.

As an environmental fungal pathogen, *Cryptococcus* neoformans lives in a broad range of ecological habitats (trees, insects, mammals, and pigeon guano, to list a few), which requires more diverse genotypes and phenotypes than fungal pathogens occupying narrower niches such as the obligate commensal *Candida albicans* [112]. The mechanisms by which *C. neoformans* generates and maintains a heterogeneous population and the impact of its life style strategies in nature on its evolutionary trajectory are poorly understood.

*C. neoformans* is a heterothallic fungus with two opposite mating types, **a** and α. Because of the preponderance of α strains, the *Cryptococcus* population is largely unisexual and thus **a**-α mating may not usually occur in nature. An exception to this might only occur in sub-Saharan Africa, where **a** isolates are more common [25],[40]. Recently, this heterothallic (outcrossing, **a**-α mating) fungus has been demonstrated to adopt a homothallic (selfing, α-α mating) lifecycle under laboratory conditions [45]. Same-sex mating also likely occurs in nature since natural αADα hybrids that are derived from two α strains of different serotypes have been identified [46]. Our finding that a significant proportion of the *Cryptococcus* population is comprised of diploid α/α isolates resulting from same-sex mating indicates that this process could be one of the driving forces for *Cryptococcus* to generate genetic diversity. Alternatively, same-sex mating could have arisen as an adaptive response to this unique largely unisexual population structure. The identification of natural α/α diploid isolates in Botswana implies that **a**-α mating and same-sex mating could co-exist in nature. The impact of same-sex mating on the *Cryptococcus* population is likely underestimated as diploids represent only one type of products generated through this life cycle. Recombinant haploid progeny could be generated from diploid intermediates, as demonstrated under laboratory conditions [45]. A previous population genetics study indicated that haploid *C. gattii* α strains generated via α-α mating could be responsible for the cryptococcosis outbreak on Vancouver Island [41]. Although sporulation so far has not been observed in these natural α/α diploids under laboratory conditions, rudimentary filaments have been observed for some α/α diploids [43]. It is conceivable that laboratory conditions may not recapitulate the natural environment where α/α diploids might sporulate (pH, temperature, humidity, nitrogen source, nutrient, and presence or absence of small molecules). Thus, the broader implications of this same-sex mating cycle remain to be fully explored and understood. Increased ploidy resulting from interspecific hybridization in unisexual forms has been shown to occur in other organisms [113]–[118]. The same-sex mating reproductive mode employed by *C. neoformans* provides an ideal paradigm to study this interesting phenomenon: lack of homogeneity in a unisexual population and the evolutionary role of a unisexual reproductive mode versus a bisexual reproductive mode.

The autodiploids represent the most common type of diploid in the natural *C. neoformans* population and they can arise via endoreplication or clonal matings. By analogy to recent studies on the parasexual cycle of *A. nidulans* [98], their function may be to generate genetic diversity *de novo* during the sexual cycle, rather than to bring together diversity from two unrelated parents. In *A. nidulans*, diploids constructed to harbor two identical genomes were found to undergo more rapid evolution, following passage and reduction to the haploid state, then isogenic haploid isolates. This parasexual process enables mutations to arise in the sheltered diploid state that exhibit inverse epistasis and therefore could not have arisen in the haploid population. This reveals that parasexual and sexual cycles, which are commonly thought to generate diversity by bringing together two genetically distinct isolates, can also generate diversity *de novo*. In this model, α/α autodiploids are produced and this is the setting in which genetic diversity arises and then is released by mitotic/parasexual or meiotic/sexual reduction from the diploid to haploid state. Future studies should test this model by addressing if the diploid state accumulates mutations (or may even be hypermutagenic) and also how the autodiploids and allodiploids complete the unisexual cycle to produce haploid spores.

Thus, application of modern genetic approaches to the questions of ploidy distribution, mechanisms of diploidization, and the effects of ploidy on the phenotypic and genotypic diversity in *Cryptococcus* will not only further our understanding of the epidemiology and pathogenesis of this important human fungal pathogen, but also holds promise for producing insight into how polyploidy facilitates evolution and adaptation in fungi and other eukaryotes.

## Materials and Methods

### Strains and growth conditions

Strains JEC21, KN99**a**, KN99α, and H99 were used as reference isolates. Natural isolates and their sources are listed in Table S1. Cells were maintained on YPD medium (1% yeast extract, 2% BactoPeptone, and 2% dextrose).

### Ploidy determination by fluorescence flow cytometry

Cells were processed for flow cytometry as described previously [45],[69]. Briefly, cells were harvested from YPD medium, washed once in PBS buffer, and fixed in 1 ml of 70% ethanol overnight at 4°C. Fixed cells were washed once with 1 ml of NS buffer (10 mM Tris–HCl (pH = 7.6), 250 mM sucrose, 1 mM EDTA (pH = 8.0), 1 mM MgCl_2_, 0.1 mM CaCl_2_, 0.1 mM ZnCl_2_) and then stained with propidium iodide (10 mg/ml) in 0.2 ml of NS buffer containing RNaseA (1 mg/ml) at 4°C for 4–16 h. Then 0.05 ml of stained cells was diluted into 2 ml of 50 mM Tris–HCl (pH 8.0) and sonicated for 1 min. Flow cytometry was performed on 10,000 cells and analyzed on the FL1 channel with a Becton–Dickinson FACScan.

### Genomic DNA purification

Strains were grown in 50 ml YPD medium at 30°C overnight with shaking. The cells were washed three times with distilled water and harvested by centrifugation at 4000×g for 8 minutes. The cell pellet was frozen immediately at −80°C, lyophilized overnight, and stored at −20°C until genomic DNA was prepared using the CTAB protocol as described previously [119].

### Determination of molecular type by AFLP

Amplified fragment length polymorphisms (AFLPs) were generated and analyzed as previously described [40]. Two different EcoRI primer combinations (EAC and ETG) were used for the selective PCR, as described previously [62]. Only intense and reproducible bands were scored to identify differences between strains.

### MLST

Three unlinked loci (*IGS1*, *PLB1*, and *GPD1*) located on different chromosomes were chosen based on a previous study showing that these loci frequently have polymorphic sites and are phylogenetically informative among serotype A populations [120]. PCR primers and amplification conditions were the same as previously described [120]. Two loci located in the mating type locus (*SXI1α*/*SXI2* ***a*** and *STE20α*/*STE20* ***a***) were chosen because they are not clustered together (>50 kb apart) and they are highly divergent. PCR primers and amplification conditions were as described previously [22],[46]. Because diploidy could be unstable for some isolates and these isolates could become aneuploid or haploid via chromosome loss or mitotic gene conversion, the initial or underlying heterozygosity might not be detected in the MLST analysis.

### Phylogenetic analysis

Phylogenetic analyses were performed with PAUP version 4.0b10 [121]. Maximum parsimony (MP) trees for the individual loci were identified with heuristic searches based on 500 random sequence additions for each data set; gaps in the sequence alignment were collapsed to a single character and included in the maximum parsimony analysis as a fifth character. Phylogenetic congruence among the *PLB1* and *GPD1* gene genealogies was tested by the partition homogeneity test with 1,000 bootstrap replicas (ILD test) implemented in PAUP [121], and no significant incongruencies were detected. MP tree for the combined gene genealogy was identified as described for the individual gene genealogies. All trees were mid-point rooted.

### Phenotypic characterization

*In vitro* phenotypic characterization was performed essentially as previously described [46],[122]. Basically, yeast cells were grown on YPD medium overnight and washed three times with water. Cell density was determined by counting using a hemacytometer and cells were serially diluted (10×). Four microliters of cells were spotted on YPD medium and incubated at 22°C for 2 days as the growth control. To analyze growth at extreme temperatures, cells were spotted on YPD medium and incubated at 4°C for 10 days and at 37°C for 2 days. To examine melanin production, cells were spotted on melanin-inducing Rose Bengal media containing L-DOPA (L-dihydroxyphenylalanine, 200 mg/L) and incubated at 22°C and 37°C in the dark for 2 days. Melanization was observed as the colony developed a dark brown color. To determine sensitivity to ultraviolet irradiation, cells were spotted on YPD medium and exposed to UV irradiation in a Stratalinker (Stratagene, La Jolla, CA) (∼48 mJ/cm^2^) for 10, 14, and 18 seconds and then incubated at 22°C for three days. To assay invasive growth, cells were spotted on SLAD medium at 30°C for five days [75].

### Microscopy

To observe capsule, yeast cells were grown on YPD medium overnight and washed three times with water. Cell density was determined by counting using a hemacytometer and cells were then transferred to Dulbecco's Modified Eagle Medium (DMEM) (Invitrogen, California) and grown for two days at 37°C. Cells were then suspended in India ink and the capsule was visualized as a white halo surrounding the yeast cell due to exclusion of ink particles. To observe morphology, yeast cells were grown on YPD and YNB medium overnight at 30°C and 37°C. Cells were then observed directly by microscopy.

### Comparative genome hybridization (CGH) and data analysis

Experiments were performed as previously described [46]. Purified genomic DNA of strains to be analyzed was sonicated to generate ∼500 bp fragments and purified with a DNA Clean and Concentrator kit (Zymo Research, CA). 5 µg of DNA was used for Cy-3 dUTP or Cy-5 dUTP labeling reactions using the Random Primer/Reaction Buffer mix (Invitrogen, BioPrime Array CGH Genomic Labeling System). Labeled DNA from the sample and the control was competitively hybridized to microarray slides containing 70-mer oligonucleotides for a *C. neoformans* whole genome and serotype- and mating-type-specific genes in the *MAT* locus (**a** and α alleles for serotypes A, B, C, and D) [46],[122]. After hybridization, arrays were scanned with a GenePix 4000B scanner (Axon Instruments, Foster City, Calif.) and analyzed using GenePix Pro v 4.0 and BRB array tools (developed by Richard Simon and Amy Peng Lam at the National Cancer Institute; http://linus.nci.nih.gov/BRB-ArrayTools.html).

### Accession Numbers

The GenBank (http://www.ncbi.nlm.nih.gov/Genbank/index.html) accession numbers for *PLB1* locus discussed in this paper are listed as follows: FJ200523: (8F.12.8.02), FJ200524 (11.97-B), FJ200525 (49F.11.97-II), FJ200526 (92-21), FJ200527 (1052), FJ200528 (bt50), FJ200529 (C24), FJ200530 (DC1033), FJ200531 (DC1035), FJ200532 (DC1054), FJ200533 (KN1), FJ200534 (KN2B5-17), FJ200535 (KN2B5-18), FJ200536 (KN2B5-19), FJ200537 (KN4B4-10), FJ200538 (KN4B7-5), FJ200539 (KN4B7-16), FJ200540 (KN24), FJ200541 (MAS93-0102), FJ200542 (MMRL743), FJ200543 (MMRL795), FJ200544 (MMRL799), FJ200545 (MMRL1088), FJ200546 (MMRL1338), FJ200547 (MMRL1368), FJ200548 (MMRL1370), FJ200549 (MMRL2445), FJ200550 (NC32-14-B), FJ200551 (NC32-14-II), FJ200552 (NC32-15-B), FJ200553 (NC32-15-II), FJ200554 (SB4), FJ200555 (TX102-14-B), FJ200556 (TX102-14-II), FJ200557 (UGB047), FJ200558 (UN20020-092), FJ200559 (UN20021-095), and FJ200560 (VPC173).

Accession numbers for *STE20* locus are list as follows: FJ200561 (8F.12.8.02), FJ200562 (49F.11.97-II), (49F.11.97-ST2) FJ200563, FJ200564 (bt2), FJ200565 (bt9), FJ200566 (bt29), FJ200567 (bt30), FJ200568 (bt50), FJ200569 (bt107), FJ200570 (bt158), FJ200571 (c92-21), FJ200572 (c20020.092), FJ200573 (c20021.095), FJ200574 (DC1033), FJ200575 (DC1035), FJ200576 (DC1052), FJ200577 (DC1054), FJ200578 (KN2B5-18), FJ200579 (KN4B7-5), FJ200580 (KN4B7-10), FJ200581 (KN5B4-1), FJ200582 (MAS92-0333), FJ200583 (MAS93-0102), FJ200584 (MMRL743), FJ200585 (MMRL795), FJ200586 (MMRL799), FJ200587 (MMRL1088), FJ200588 (MMRL1368), FJ200589 (MMRL1370), FJ200590 (MMRL2445), FJ200591 (NC32-14-B), FJ200592 (NC32-14-II), FJ200593 (NC32-15-B), FJ200594 (NC32-15-II), FJ200595 (SB4), FJ200596 (TX102-14-B), FJ200597 (TX102-14-II), FJ200598 (UBG047), and FJ200599 (VPC173).

Accession numbers for *GPD1* locus are list as follows: FJ200600 (8F.12.8.02), FJ200601 (49F.11.97-B), FJ200602 (49F.11.97-II), FJ200603 (bt29), FJ200604 (bt30), FJ200605 (bt50), FJ200606 (C24), FJ200607 (DC1033), FJ200608 (DC1035), FJ200609 (DC1052), FJ200610 (DC1054), FJ200611 (DC20020-092), FJ200612 (KN1), FJ200613 (KN2B5-17), FJ200614 (KN2B5-18), FJ200615 (KN2B5-19), FJ200616 (KN4B4-10), FJ200617 (KN4B7-5), FJ200618 (KN4B7-16), FJ200619 (KN24), FJ200620 (MAS092-0333), FJ200621 (MAS93-0102), FJ200622 (MMRL743), FJ200623 (MMRL795), FJ200624 (MMRL799), FJ200625 (MMRL1088), FJ200626 (MMRL1338), FJ200627 (MMRL1368), FJ200628 (MMRL1370), FJ200629 (MMRL2445), FJ200630 (NC32-14-B), FJ200631 (NC32-14-II), FJ200632 (NC32-15-II), FJ200633 (NC32-15-B), FJ200634 (SB4), FJ200635 (TX102-14-B), FJ200636 (TX102-14-II), FJ200637 (UG047), FJ200638 (UN20021-095), and FJ200639 (VPC173).

## Supporting Information

Figure S1 Comparative genome hybridization of the diploid isolates 102-14, MMRL2445, MMRL799, and 1033. Genomic DNA from the selected environmental and clinical diploid isolates and the control strain H99 (Aalpha) was fragmented, labeled with fluorescent dyes, and competitively hybridized to a 70-mer genomic microarray. The fluorescent signal intensity was normalized across the genome. The log2 value of the fluorescence intensity ratios for the serotype A alpha specific alleles between sample/H99 are shown. Black solid bars are for strain 102-14, open bars are for strain MMRL2445, bars with lines are for strain MMRL799, and grey bars are for strain 1033. A schematic representation of the serotype A alpha mating type locus is illustrated at the bottom [65]. Blue color indicates intergenic regions and yellow color indicates highly conserved genes.
(0.48 MB TIF)

Figure S2 Phylogenetic tree using *IGS1*. Representative one out of eight MP trees inferred from the *IGS1* locus.
(1.08 MB TIF)

Table S1 Strains used
(0.11 MB XLS)
