# Supplementary figures and images for: Diploids in the Cryptococcus neoformans Serotype A Population Homozygous for the α Mating Type Originate via Unisexual Mating
Source: PLoS Pathog. 2009 Jan 30;5(1):e1000283. doi: 10.1371/journal.ppat.1000283 (PMC2629120; doi:10.1371/journal.ppat.1000283)

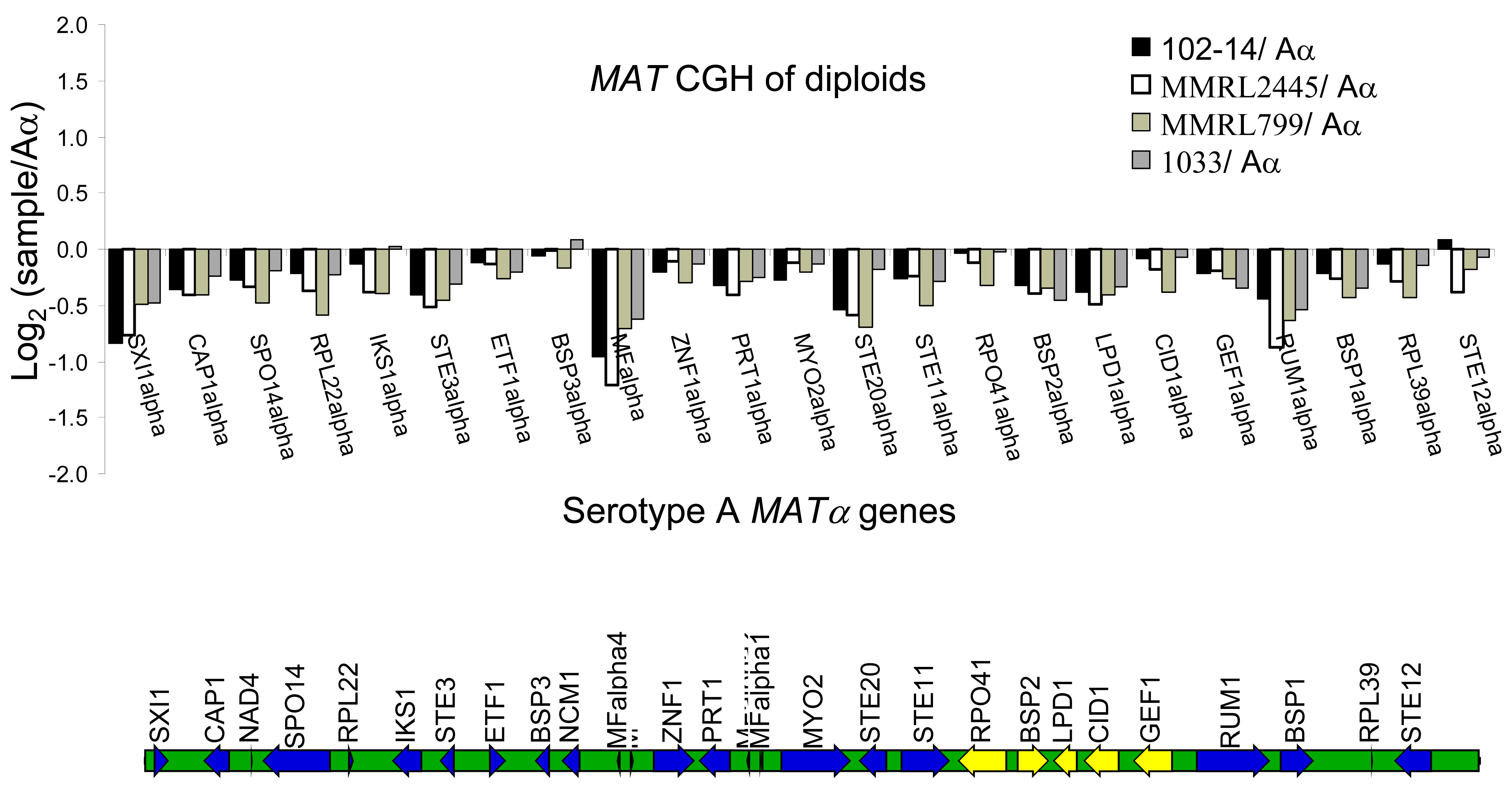

Supplement: Figure S1 — Comparative genome hybridization of the diploid isolates 102-14, MMRL2445, MMRL799, and 1033. Genomic DNA from the selected environmental and clinical diploid isolates and the control strain H99 (Aalpha) was fragmented, labeled with fluorescent dyes, and competitively hybridized to a 70-mer genomic microarray. The fluorescent signal intensity was normalized across the genome. The log2 value of the fluorescence intensity ratios for the serotype A alpha specific alleles between sample/H99 are shown. Black solid bars are for strain 102-14, open bars are for strain MMRL2445, bars with lines are for strain MMRL799, and grey bars are for strain 1033. A schematic representation of the serotype A alpha mating type locus is illustrated at the bottom [65]. Blue color indicates intergenic regions and yellow color indicates highly conserved genes. (0.48 MB TIF) [file ppat.1000283.s001.tif]

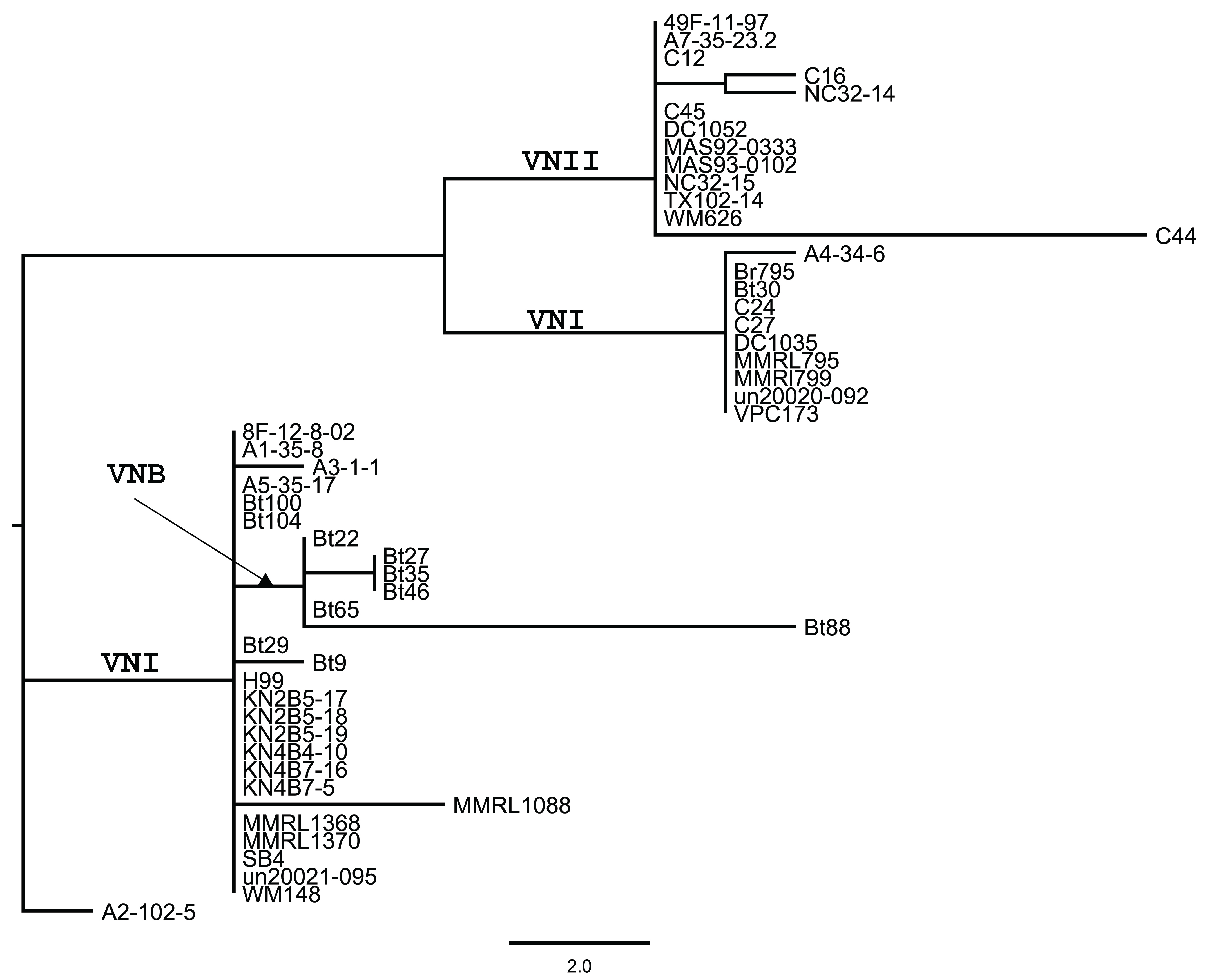

Supplement: Figure S2 — Phylogenetic tree using IGS1. Representative one out of eight MP trees inferred from the IGS1 locus. (1.08 MB TIF) [file ppat.1000283.s002.tif]
